# Supplementary material for: Monitoring real-time transmission heterogeneity from incidence data
Source: PLoS Comput Biol. 2022 Dec 1;18(12):e1010078. doi: 10.1371/journal.pcbi.1010078 (PMC9746975; doi:10.1371/journal.pcbi.1010078)
Supplement: S1 Text — (PDF) [file pcbi.1010078.s001.pdf]

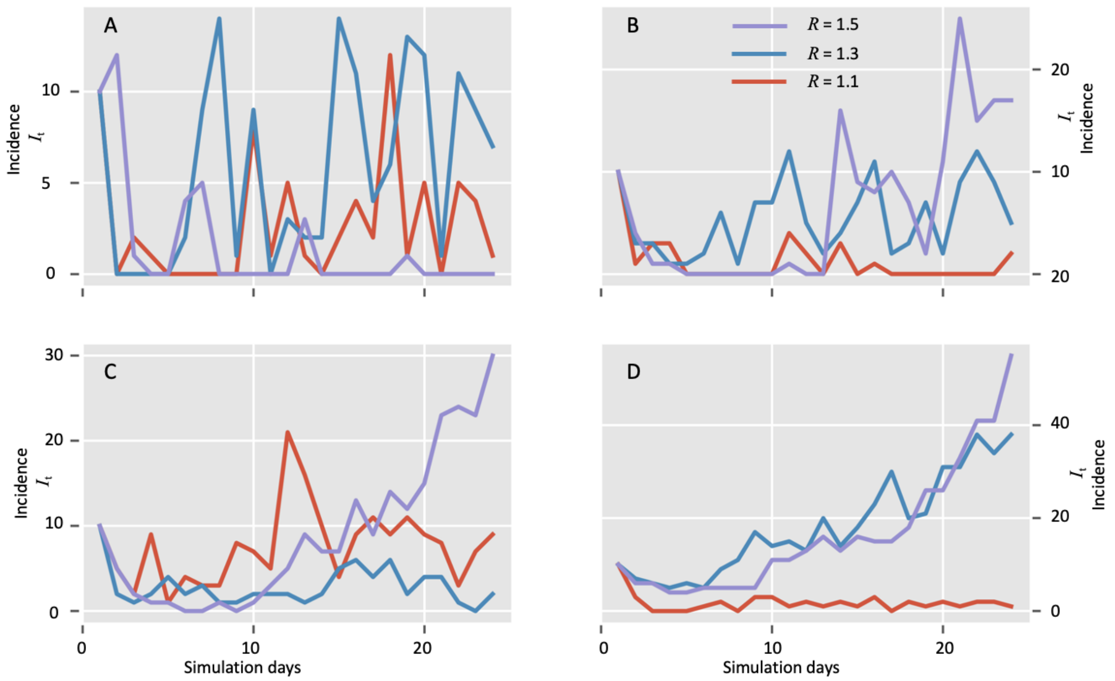

Figure A: **Typical simulated incidence data.** Each simulation begins with 10 infections and continues for 24 days. The incidence data at each day was generated according to the instant-individual heterogeneity model. Color lines showed the simulated incidence data under different simulation settings (i.e., reproduction number R and dispersion number k). A. $k = 0.2$; B. $k = 0.5$; C. $k = 2$; D. $k = 5$.

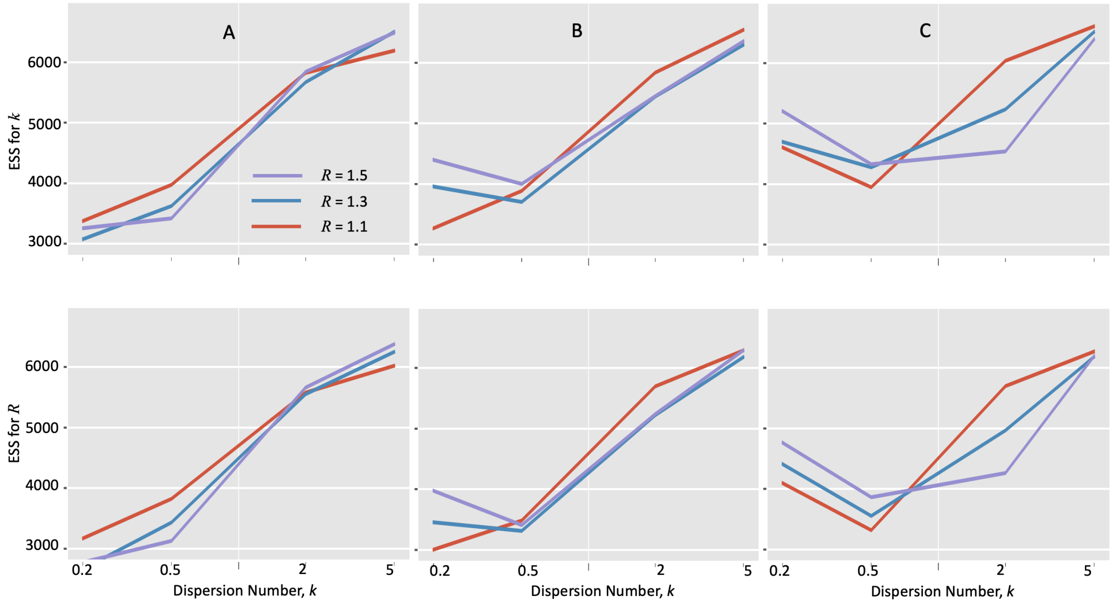

Figure B: **The effective sample size (ESS) of parameter estimation with the instant-individual heterogeneity model in simulation analysis.** Color lines showed the mean of ESS for a parameter estimate over 100 simulations under different settings (i.e., reproduction number R and dispersion number k). A, B, and C. Estimation with daily reported incidence data of different time lengths, i.e., window size = 7 days (A), 14 days (B), and 21 days (C).

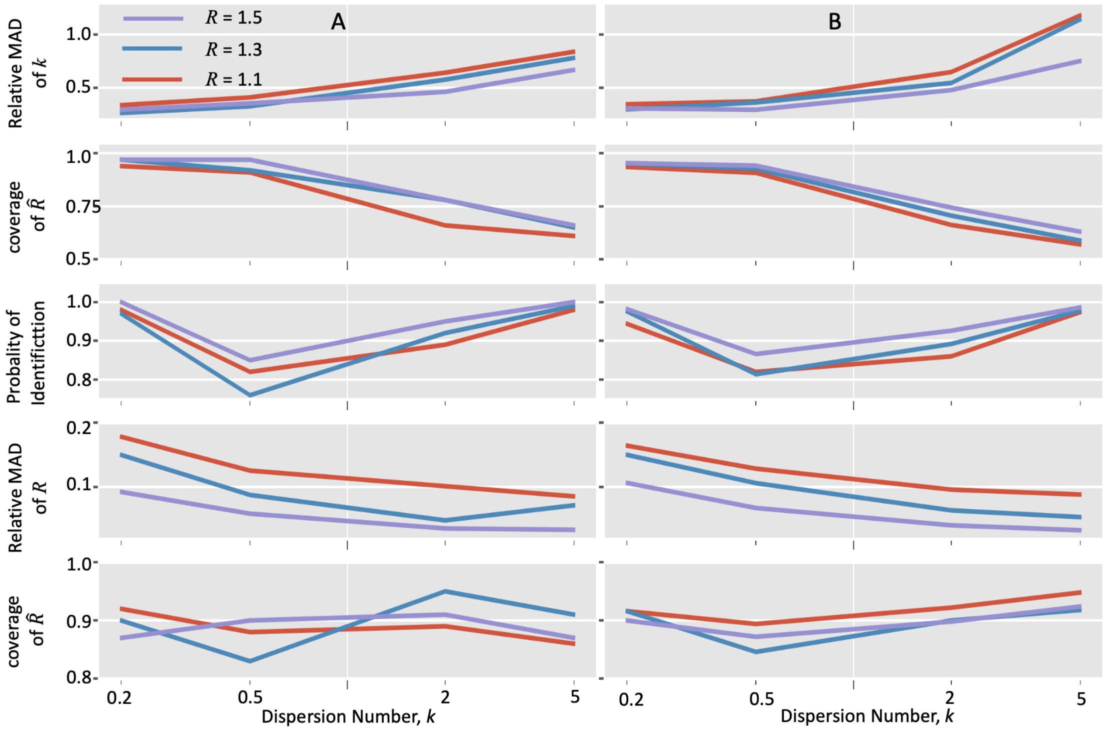

Figure C: **Performance comparison of the instant-individual heterogeneity model with different simulation runs.** Each simulation began with 10 cases and stopped at 24 days. Incidence data at each simulation day were generated with the instant-individual heterogeneity model with given reproduction number and dispersion number. The data in the last period of 21 days were used to estimate the transmission dynamics. The relative median absolute deviation (MAD)and the coverage of 95% high probability density interval were calculated for the estimation of reproduction number R and dispersion number k respectively. The probability of identification (defined in the section of methods) was also calculated for the estimation of dispersion number k. A. Performance under 100 simulation runs; B. Performance under 500 simulation runs.

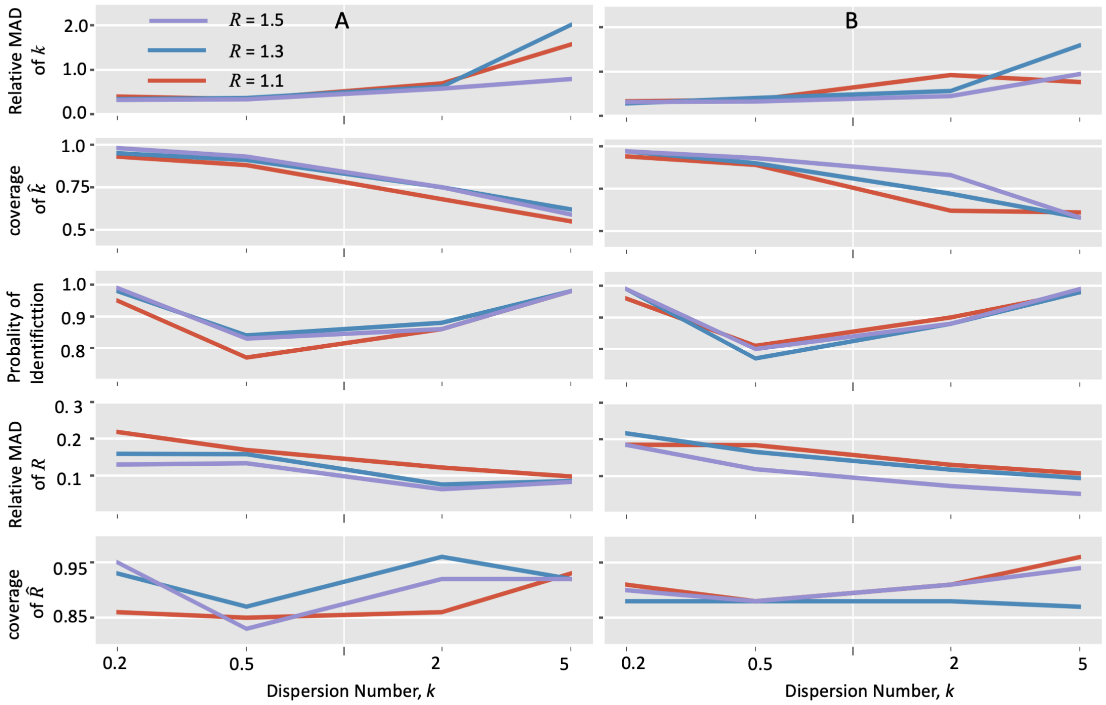

Figure D: **Performance comparison of the instant-individual heterogeneity model with different methods of simulation.** A. Individual-level simulation; B. Population-level simulation. Under the former condition, the offspring of each index was simulated as equation (6) in the main text and then was aggregated to generate the incidence. Under the later condition, the incidence data were directly generated according equation (3) in the main text. Under both conditions, each simulation began with 10 cases and stopped at 24 days. The data in the last period of 21 days were used to estimate the transmission dynamics. The relative median absolute deviation (MAD) and the coverage of 95% high probability density interval were calculated for the estimation of the reproduction number R and the dispersion number k respectively. The probability of identification (defined in the Methods section) was also calculated for the estimation of dispersion number k.

Table A: **Comparison of new dispersion number estimates with earlier records.**

Epidemic	Estimate of dispersion number with the Instant-individual heterogeneity model	Record of dispersion number	Overlapped
COVID-19, Hong Kong, 2020	0.19 (95%HPD: 0.13∼0.26)	0.43 (95%CI: 0.29∼0.67)[1]	No
COVID-19, Hong Kong, 20-21	0.16 (95%HPD: 0.14∼0.19)	0.2 (95%CI: 0.16∼0.25)[2]	Yes
COVID-19, Tianjin, 2020	0.51 (95%HPD: 0.16∼1.55)	0.25 (95%CI: 0.13∼0.88)[3]	Yes
COVID-19, Georgia, 2020	0.023 (95%HPD: 0.016∼0.031)	0.009 (95%CI: 0.007∼0.348)[4]	Yes
MERS, South Korea	0.1 (95%HPD: 0.056∼0.17)	0.06 (95%CI: 0.05∼0.08)[5]	Yes
Ebola, Sierra Leone	0.065 (95%HPD: 0.037∼0.11)	0.18 (95%CI: 0.1∼0.26)[6]	Yes
Measles, Canada	0.94 (95%HPD: 0.26∼5.60)	90%CI: 0.12∼0.65)[7]	Yes

References

[1] Adam DC, Wu P, Wong JY, Lau EH, Tsang TK, Cauchemez S, et al. Clustering and superspreading potential of SARS-CoV-2 infections in Hong Kong. Nature Medicine. 2020;26(11):1714–1719.

[2] Adam D, Gostic K, Tsang T, Wu P, Lim WW, Yeung A, et al. Time-varying transmission heterogeneity of SARS and COVID-19 in Hong Kong. 2022;.

[3] Zhang Y, Li Y, Wang L, Li M, Zhou X. Evaluating transmission heterogeneity and super-spreading event of COVID-19 in a metropolis of China. International journal of environmental research and public health. 2020;17(10):3705.

[4] Wang J, Chen X, Guo Z, Zhao S, Huang Z, Zhuang Z, et al. Superspreading and heterogeneity in transmission of SARS, MERS, and COVID-19: A systematic review. Computational and Structural Biotechnology Journal. 2021;19:5039–5046.

[5] Chowell G, Abdirizak F, Lee S, Lee J, Jung E, Nishiura H, et al. Transmission characteristics of MERS and SARS in the healthcare setting: a comparative study. BMC medicine. 2015;13(1):1–12.

[6] Althaus CL. Ebola superspreading. The Lancet Infectious Diseases. 2015;15(5):507–508.

[7] Lloyd-Smith JO, Schreiber SJ, Kopp PE, Getz WM. Superspreading and the effect of individual variation on disease emergence. Nature. 2005;438(7066):355–359.
