# Supplementary material for: Monitoring real-time transmission heterogeneity from incidence data
Source: PLoS Comput Biol. 2022 Dec 1;18(12):e1010078. doi: 10.1371/journal.pcbi.1010078 (PMC9746975; doi:10.1371/journal.pcbi.1010078)
Supplement: Attachment — Submitted filename: Monitoring_real_time_transmission_heterogeneity_from_Incidence_data_Referee_Report.pdf [file pcbi.1010078.s002.pdf]

Zhang et al. propose a method to calculate the heterogeneity in the offspring distribution through quantification of the dispersion parameter, k, from summarized incidence. The ability to accurately quantify over-dispersion from this type of epidemiological data would be a major advance, providing a substantially easier method to calculate over-dispersion. They find that their method produces different estimates of R_t than a more standard model and identify substantial over-dispersion in the offspring distribution in the settings they investigate.

While the authors apply their method to simulated data and to incidence data from a range of settings, I have a number of concerns about their method and results. The authors comparison of R_t and k to published estimates from alternative methods is limited. They do not examine the sensitivity of their results to added variance from other mechanisms, such as irregular reporting. Some of the estimated values of k are also very low – orders of magnitude lower than many common estimates for SARS-CoV-2. With the evidence given, it's hard to tell when this method may be appropriately applied or trusted to produce accurate results.

1 Major Comments

- The authors do not formally compare their results to estimates from the same time period and settings, such as Lau 2020. However, the estimated values of k from Lau 2020 are on the order of 10^{-1} rather than Zhang et al.'s estimates which seem to be somewhere between 10^{-1} and 10^{-2}. Why does this difference occur and does it similarly occur in other settings?
- Incidence data frequently have sources of variation other than due to variation in transmission. How robust is this method to these other sources of variation?
- The authors pick very wide "non-informative" priors. Can the sensitivity of the results across a range of prior specifications be checked? Particularly for some the more common types of "weakly informative" priors (see e.g. https://www.mdpi.com/1099-4300/19/10/555/htm)
- In the simulation study, the results for the dispersion parameter, k, are only reported with respect to its point estimate and if it is above or below 1. Can the authors report the HPD for k and the whether the model appropriately recovers the simulation parameter across the specifications?
- Between lines 119 and 120, the equation $\Lambda_t \eta_t \sim \Gamma(k_t, \frac{k_t}{\Lambda_t R_t})$ seems to imply that Λ_t is distributed in part according to Λ_t. Can the authors clarify or modify their notation?
- The relative RMSE of k is very high when k grows larger. Can the authors explain why this is occurring?
- The estimated values for k from the Omicron pandemic are astoundingly low – on the order of 10^{-4}. Can the authors add additional supplemental

detail and discussion on this result, especially in comparison to related values in the literature?

- The simulations were only repeated 100 times and the results seem as if they may be noisy (MCSE is high). Can the authors run a much higher number of Monte Carlo simulations?
- While there is code available on Github, it doesn't seem to have the code to perform the simulation study or produce all of the figures. Only the code for Figures 2, 3, and 4 seems to be available.
- In general, it's hard to tell how specifically the simulation study was coded. Was an individual-level branching process generated and then the incidence summed at each time step? Or was incidence generated on the aggregated scale and variance simulated around the mean?
- The authors do not report the incidence curves for their simulated results. How many individuals are infected in the simulation and is it comparable to the settings with real data?
- In general, it's hard to tell from these results when the method is appropriate to apply and when the authors believe it would be inappropriate. Can the authors expand on their limitations to explain when this method should not be applied or when it would fail to perform well?
- The authors don't report convergence diagnostics for the Bayesian models.

2 Minor Comments

- In Equation (1), it is unclear in $\bar{I}_1^{t-1}$ the role of the superscript and the subscript are.
- I'm not sure what the phrase "evolutionary processes occur on the same timescale as epidemiological processes" (line 26) means.
- Likewise, I'm not sure how to understand "In other words, the standard model is identical to assume $v^i_{s,t} = w_{ts}R_t$" (line 115).
- I also have trouble following lines 465-469.
- In Figure 3, it's hard to understand the x axis.
- In Figure 5, it's hard to tell what's happening in the plots of k because the plots are log scaled and the credible intervals are so wide.
- On line 379, "The underlying reason for this decrease in Rt was the deploying of control measures by the South Africa government as indicated by the government stringency index." This statement seems overconfident in ascribing a mechanism behind the decrease.
