# Supplementary material for: Monitoring real-time transmission heterogeneity from incidence data
Source: PLoS Comput Biol. 2022 Dec 1;18(12):e1010078. doi: 10.1371/journal.pcbi.1010078 (PMC9746975; doi:10.1371/journal.pcbi.1010078)
Supplement: Attachment — Submitted filename: Zhang et al.pdf [file pcbi.1010078.s003.pdf]

Re: Monitoring real-time transmission heterogeneity from Incidence data

The paper by Zhang et al. extends previously established methods for estimating Rt to include individual transmissibility, with the aim of providing a simple method of co-estimating real-time transmissibility and its heterogeneity (using only incidence data).

Overall, this is a clearly written manuscript and I enjoy reading it.

I have three major comments/suggestions:

(1) The authors perform simulation studies to validate their approach. Specifically, they simulated incidence data from their proposed model (a branching process essentially) and showed the key model parameters of interested can be estimated reasonably well. I think this is a good test but sufficient. A much stronger test would be to simulate the epidemic using an individual-level S-I-R type model (a model that is more representative of the real transmission process), and fit their model to the incidence data from the simulation. Of course, parameter like the dispersion parameter like k_t will not longer be explicit in an S-I-R type model, but the authors can compute (and compare) empirical k_t as a summary statistics from the simulated epidemic.

(2) I would like to see more in the sensitivity analysis, particularly, on time-varying reporting rate. In its current set of sensitivity analysis, only constant reporting rate is tested. This may be true for pathogens like flu, but I imagine this assumption may break down for novel pathogens like SARS-CoV-2. A time-varying reporting rate will likely to have a large impact on their model estimates, given that their inference largely depends on inputs of incidence data (Equation 4). If this turns out to be case, such sensitivity towards time-varying reporting rate should be reported.

(3) In contrast to a general spatio-temporal transmission model, their approach considers only the temporal aspect of the transmission. I would like the authors to discuss more on how potential spatial heterogeneity of transmissibility may impact their results, and make related caveats clearer in the discussion.
