# Supplementary material for: Monitoring real-time transmission heterogeneity from incidence data
Source: PLoS Comput Biol. 2022 Dec 1;18(12):e1010078. doi: 10.1371/journal.pcbi.1010078 (PMC9746975; doi:10.1371/journal.pcbi.1010078)
Supplement: Attachment — Submitted filename: Zhang_et_review__2.pdf [file pcbi.1010078.s005.pdf]

In general, I think the manuscript has improved due to the thoughtful revisions of the authors. In particular, the analysis seems more robust to me and the comparison to the literature is more clear. I also appreciate the authors' careful and thoughtful responses to the reviewer feedback. Furthermore, the authors' general result that the more flexible IIH modelis better at fitting incidence data is a useful one and, I think, convincing.

However, these careful responses and additional analyses have made clearer to me some concerns with the manuscript. These fall into three general categories:

1. Concerns that the estimates of k are not particularly close to their comparisons from the literature. In Lau et al., the estimates of k are on the order of 0.5 and about 0.25 at their lowest in Dougherty County (see Figure 2A) [1]. These numbers are quite different than Zhang et al.'s estimate of 0.016 (95% HPD: 0.01, 0.023). The estimated k from the MERS case study presented in Figure 2A also seems quite different to me. I don't find these comparisons particularly convincing that the IIH method is accurately recovering the same k as the standard method using individual level contact tracing data. It may the case that the IIH method works appropriately, but these comparisons need to be reworked to make that case as well as explain when and where the method fails. As an aside, I couldn't find $k \approx 0.009$ anywhere in Lau at al. and the manuscript's reference should be corrected or clarified.

2. Concerns that the simulation approach is inappropriate. As best I can tell, the mock data simulates I_t from a negative binomial distribution with mean $R_t \Lambda_t$ and some fixed k and the IIH method recovers that R_t and k. But to show that the IIH method can replace the approach using individual level data, the simulations need to show that the method can recover k when I_t is the sum of *individual level realizations* from a negative binomial distribution (i.e., $I_t = \sum_{n=1}^{N} \eta_{nt}; \eta_{nt} \sim NB(R_t, k)$). These individual-level simulations should be, at minimum, a branching process although a network-based approach would be stronger I think. Showing only that the IIH method can recover k with population-level parametric noise is not enough to illustrate that the method can estimate individual-level transmission heterogeneity. Any simulations examining the impact of the other sources of heterogeneity should be added on top of this individual-level framework.

3. The estimates of k presented in this manuscript are consistently much lower than the generally accepted values in the literature. The literature around SARS-CoV-2 has settled around k in roughly in the range of $0.1 - 0.3$ [2, 3, 4]. These estimates correspond to somewhere between 10%-20% of cases leading to the bulk of transmission and roughly 50% of cases leading to at least one additional case. The estimated values of k in this manuscript roughly correspond to 5% of primary cases producing at least one secondary case. These are *substantially* different paradigms and, in

my reading, would be a sharp departure from the literature on SARS-CoV-2. These differences should be more closely examined, if they can be adequately justified.

Because of these concerns, I am not convinced by the main finding: that the IIH approach accurately estimates of individual-level overdispersion in the secondary case distribution. While this may be true, I would need to see the manuscript substantially reworked to address the above concerns before I could recommend it for publication.

References

[1] Max SY Lau, Bryan Grenfell, Michael Thomas, Michael Bryan, Kristin Nelson, and Ben Lopman. Characterizing superspreading events and age-specific infectiousness of sars-cov-2 transmission in georgia, usa. *Proceedings of the National Academy of Sciences*, 117(36):22430–22435, 2020.

[2] Akira Endo, Sam Abbott, Adam J Kucharski, Sebastian Funk, et al. Estimating the overdispersion in covid-19 transmission using outbreak sizes outside china. *Wellcome open research*, 5, 2020.

[3] Dillon C Adam, Peng Wu, Jessica Y Wong, Eric HY Lau, Tim K Tsang, Simon Cauchemez, Gabriel M Leung, and Benjamin J Cowling. Clustering and superspreading potential of sars-cov-2 infections in hong kong. *Nature Medicine*, 26(11):1714–1719, 2020.

[4] Ashish Goyal, Daniel B Reeves, E Fabian Cardozo-Ojeda, Joshua T Schiffer, and Bryan T Mayer. Viral load and contact heterogeneity predict sars-cov-2 transmission and super-spreading events. *Elife*, 10:e63537, 2021.
