# Supplementary material for: Monitoring real-time transmission heterogeneity from incidence data
Source: PLoS Comput Biol. 2022 Dec 1;18(12):e1010078. doi: 10.1371/journal.pcbi.1010078 (PMC9746975; doi:10.1371/journal.pcbi.1010078)
Supplement: Attachment — Submitted filename: re2letter1024.pdf [file pcbi.1010078.s006.pdf]

We are grateful to the valuable comments from the Editor and the reviewer. In the following, we provide a detailed report on how we revised the manuscript in response to the comments and suggestions. We made a substantial revision to the manuscript. Beside revising according to the reviewer’s comments, we have corrected some typos and similar minor changes. New words are blue and the deleted words appear as crossed over red text.

1. Editor’s comment

Given the continued discrepancy between published estimates of "k" and those presented in this paper, I agree with R1's updated assessment and R2's prior assessment that individual-level simulations with known "k" (or at least comparable measure of super-spreading) are a necessity for this work. Without such individual-level simulations, it's simply not possible to evaluate the methodology nor is it easy to interpret the substantial differences in "k" parameters estimated in this paper vs. those reported in prior works. While I appreciate the authors' attention to the past round of reviews and verbal explanation for what may be driving the difference in "k" parameter estimates, more quantitative work is needed to both demonstrate the validity of the approach and mechanistically account for the reported discrepancies.

Response: *Thanks for these valuable suggestions to improve this study. We conducted an individual-level simulation to generate the incidence data in a different way (line 190~204) and then estimated the dispersion number ”k” with the proposed model. The resulting estimation was comparable with our previous simulation studies (line 348~352).*

To make a complete comparison between our estimates of heterogeneity and the records in literature, we calculated the overall dispersion number for seven epidemics involved in this study (Table1 in this letter). Estimates for five epidemics were consistent with the records in terms of overlapped interval estimation. The estimates for other two exceptional were also close to the records.

Additionally, your comment that our estimates differed from published estimates made us check our program in great detail and a bug was found. We fixed the bug in our python code and recalculated all the results for historic epidemic data. Generally, there were some changes in the estimation of heterogeneity. And the discrepancy between our estimates and the records decreased after fixing the bug. For the 6 out of 7 historic epidemics, our estimates of dispersion number were consistent with the published results (Table1 in this letter, also line 398~403 and Table S1 in the revised manuscript). As to the exceptional example, our estimates also fell within the reference range of the heterogeneity of SARS-CoV-2 (line 401~403).

As pointed out by Wang et al [2]*, the estimates of k for a particular epidemic may vary because of differences in study design and the estimation methods. It is important to identify the heterogeneous transmission pattern for an outbreak. Our model successfully identified the heterogeneity for all these epidemics.*

The incorrect code and the analyzed datasets have been publicly available since the first submission. The new codes are also available online for others to use and check. (https://github.com/yunPKU/infer_heterogeneity_from_incidence). We can of course not be certain it has no further bugs, but now it has been scrutinized in detail so the risk is at least much smaller.

Explanation about the bug in the python code:

***Where is the bug**:*

The bug lies in the first line in the function of "gamma2discrete" which calculates the probability distribution of serial interval (defined in line 94~97). The input parameters include the mean and standard deviation of the serial interval (denoted as mean_GT and sd_GT respectively) and the maximum length of infectious period for a particular case (denoted as MaxInfctPrd).

We modelled the serial interval as a gamma shaped distribution, so we firstly need to transform the mean and standard deviation of the distribution into scale and rate parameters.

What is the bug:

*The wrong code was: shape_para = mean_GT/sd_GT**2*

*The corrected code was: shape_para = (mean_GT/sd_GT)**2*

(https://en.wikipedia.org/wiki/Gamma_distribution for the details of transformation)

The changes in the estimation

As list in Table 1 of this letter, there were some changes in the estimates of heterogeneity after fixing the bug. This is reasonable because the bug is almost equal to assign a wrong mean and a wrong standard deviation for the serial interval used in analysis. Based on our sensitivity analysis (line 470, subsection of sensitivity analysis), our estimate of heterogeneity was robust to the bias either in the mean or in the standard deviation of the serial interval. Therefore, the estimation of heterogeneity changes little after fixing the bug.

2. Reviewer #1

In general, I think the manuscript has improved due to the thoughtful revisions of the authors. In particular, the analysis seems more robust to me and the comparison to the literature is more clear. I also appreciate the authors' careful and thoughtful responses to the reviewer feedback. Furthermore, the authors' general result that the more flexible IIH modelis better at fitting incidence data is a useful one and, I think, convincing.

Response: *Thank you for the positive feedback!*

However, these careful responses and additional analyses have made clearer to me some concerns with the manuscript. These fall into three general categories:

1. Concerns that the estimates of k are not particularly close to their comparisons from the literature. In Lau et al., the estimates of k are on the order of 0.5 and about 0.25 at their lowest in Dougherty County (see Fig- ure 2A) [1]. These numbers are quite different than Zhang et al.'s estimate of 0.016 (95% HPD: 0.01, 0.023). The estimated k from the MERS case study presented in Figure 2A also seems quite different to me. I don't find these comparisons particularly

convincing that the IIH method is accurately recovering the same k as the standard method using individual level contact tracing data. It may the case that the IIH method works appropriately, but these comparisons need to be reworked to make that case as well as explain when and where the method fails. As an aside, I couldn't find k ≈ 0.009 anywhere in Lau at al. and the manuscript's reference should be corrected or clarified.

Response: *Thanks for your careful evaluation of our results. We made a complete comparison between our estimates of heterogeneity and the records in literature for all the seven epidemics involved in this study (Table 1 in this letter). Generally, the estimates for five epidemics were consistent with the records, and the estimates for other two exceptional were also close to the records.*

Also, your comments on the discrepancy between our results and the records made us check the python code in great detail and a bug was found (the above explanation about the bug). We fixed the bug and recalculated the results for all the historic epidemics. For six out of seven historic epidemics, our new estimates were consistent with the records (Table 1 in this letter, line 389~403). Particularly, the new estimation for the MERS epidemic was 0.10 (95% HPD: 0.056~0.17) (line 378~381), which was consistent with the records of 0.06(95% CI: 0.05~0.08) in literature [1].

The records of k ≈ 0.009 (95%CI: 0.007~0.348) for the epidemic of COVID-19 in Georgia, USA were provided by Wang et al who re-estimated the dispersion number on the basis of Lau's data [2]. We clarified the reference for these records (line 435). Our estimation of "k" for this epidemic was 0.023 (95%HPD: 0.016~0.031), which overlapped with the records (line 433).

2. Concerns that the simulation approach is inappropriate. As best I can tell, the mock data simulates I_t from a negative binomial distribution with mean $R_t\Lambda_t$ and some fixed k and the IIH method recovers that R_t and k. But to show that the IIH method can replace the approach using individual level data, the simulations

need to show that the method can recover k when I_t is the sum of individual level realizations from a negative binomial distribution (i.e., $I_t = \sum_{n=1}^{N} \eta_{nt}; \eta_{nt} \sim NB(R_t, k)$). These individual- level simulations should be, at minimum, a branching process although a network-based approach would be stronger I think. Showing only that the IIH method can recover k with population-level parametric noise is not enough to illustrate that the method can estimate individual-level transmission heterogeneity. Any simulations examining the impact of the other sources of heterogeneity should be added on top of this individual- level framework.

Response: *Thanks for your valuable suggestion on the individual level simulation. We conducted an individual-level simulation to generate the incidence data (line 190~204). The resulting estimation was comparable with our previous studies based on population level of simulation (line 348~352).*

3. The estimates of k presented in this manuscript are consistently much lower than the generally accepted values in the literature. The literature around SARS-CoV-2 has settled around k in roughly in the range of 0.1 − 0.3 [2, 3, 4]. These estimates correspond to somewhere between 10%-20% of cases leading to the bulk of transmission and roughly 50% of cases leading to at least one additional case. The estimated values of k in this manuscript roughly correspond to 5% of primary cases producing at least one secondary case. These are substantially different paradigms and, in my reading, would be a sharp departure from the literature on SARS- CoV-2. These differences should be more closely examined, if they can be adequately justified.

Response: *Thanks for pointing it out! We estimated the dispersion numbers for four COVID-19 epidemics, including in Hongkong,2020, in Tianjin, 2020, in Hongkong, 2020~2021, in USA 2020. The estimates for the last three epidemics were consistent with the records in literatures (line 398~403, table S1). The estimate for the first epidemic was 0.19 which fell into the reference range of 0.1~0.3 (line 401~403).*

Because of these concerns, I am not convinced by the main finding: that the IIH approach accurately estimates of individual-level overdispersion in the secondary case distribution. While this may be true, I would need to see the manuscript substantially reworked to address the above concerns before I could recommend it for publication.

Response: *Thanks for these valuable suggestions! We conducted an individual-level simulation study, fixed a bug in the python code, and re-calculated all the results in the manuscript. Please see the highlighted text the manuscript.*

As pointed out by Wang et al [2]*, the estimates of k for a particular epidemic may vary because of differences in study design and the estimation methods. It is important to identify the heterogeneous transmission pattern for an outbreak. Our model successfully identified the heterogeneity for all these epidemics.*

Table 1 Comparison of dispersion number estimation with records in literatures.

Epidemic	Published results Mean (95%CI)	Estimation before fixing the bug MAP (95%HPD)	Overlapped	Estimation after fixing the bug MAP (95%HPD)	Overlapped
COVID-19, HongKong, 2020	0.43 (0.29, 0.67) [3]	0.3 (0.2~0.43)	Yes	0.19 (0.13, 0.26）	Close
COVID-19, HongKong ,2020~2021	0.2 (0.16, 0.25) [4]	0.21 (0.17,0.24)	Yes	0.16 (0.14,0.19)	Yes
COVID-19, Tianjin, 2020	0.25 (0.13, 0.88) [5]	0.54 (0.16~1.33)	Yes	0.51 (0.16， 1.55)	Yes
COVID-19, Georgia, 2020	0.009 (0.007,0.348) [2]	0.016 (0.01~0.023)	Yes	0.023 (0.016， 0.031)	Yes
MERS, South Korea	0.06 (0.05, 0.08) [1]	0.3 (0.12~0.57)	No	0.1 (0.056, 0.17)	Yes

Ebola, Sierra Leone	0.18 (0.1,0.26) [6]	0.062 (0.035, 0.099)	Very close	0.065 (0.037,0.11)	slightly overlapped
Measles, Canada	90%CI (0.12,0.65) [7]	1.44 (0.52, 88.8)	Yes	0.94 (0.26,5.60)	Yes

Reference

[1] Chowell G, Abdirizak F, Lee S, Lee J, Jung E, Nishiura H, et al. Transmission characteristics of MERS and SARS in the healthcare setting: a comparative study. BMC medicine. 2015;13(1):1–12.

[2] Wang J, Chen X, Guo Z, Zhao S, Huang Z, Zhuang Z, et al. Superspreading and heterogeneity in transmission of SARS, MERS, and COVID-19: A systematic review. Computational and Structural Biotechnology Journal. 2021;19:5039–5046.

[3] Adam DC, Wu P, Wong JY, Lau EH, Tsang TK, Cauchemez S, et al. Clustering and superspreading potential of SARS-CoV-2 infections in Hong Kong. Nature Medicine. 2020;26(11):1714–1719.

[4] Adam D, Gostic K, Tsang T, Wu P, Lim WW, Yeung A, et al. Time-varying transmission heterogeneity of SARS and COVID-19 in Hong Kong. 2022;

[5] Zhang Y, Li Y, Wang L, Li M, Zhou X. Evaluating transmission heterogeneity and super-spreading event of COVID-19 in a metropolis of China. International journal of environmental research and public health. 2020;17(10):3705.

[6] Althaus CL. Ebola superspreading. The Lancet Infectious Diseases. 2015;15(5):507–508.

[7] Lloyd-Smith JO, Schreiber SJ, Kopp PE, Getz WM. Superspreading and the ef- fect of individual variation on disease emergence. Nature. 2005;438(7066):355– 359.
