# Supplementary material for: Monitoring real-time transmission heterogeneity from incidence data
Source: PLoS Comput Biol. 2022 Dec 1;18(12):e1010078. doi: 10.1371/journal.pcbi.1010078 (PMC9746975; doi:10.1371/journal.pcbi.1010078)
Supplement: Attachment — Submitted filename: ResponseLetter0729.docx [file pcbi.1010078.s004.docx]

We are grateful to the valuable comments from the Editor, the Associate Editor and the two reviewers. In the following, we provide a detailed report on how we revised the manuscript in response to the comments and suggestions.

**Editor’s comments**

I agree with both reviewers that this is a potentially interesting approach and covers a very timely topic in the field of computational epi, i.e., the estimation of the degree of overdispersion in secondary cases during an epidemic. However, both reviewers identified a number of substantive points that must be thoroughly addressed during the revision process. In particular, I suggest that the authors pay close attention to R1's detailed comments. Please also note that both reviewers highlighted the need for expanded sensitivity analyses and simulations to demonstrate the validity of the approach. Addressing the need for improved sensitivity analyses and simulation modeling will be a critical part of successfully revising. In addition, R1 noted that many of the estimates of the over-dispersion parameter, "k", deviate from what appears to be accepted in the literature. This is not necessarily a problem, but warrants quite a bit more explanation/justification. Beyond the reviewer comments, I might also suggest including an analysis of at least one other pathogen where the over-dispersion parameter has been widely estimated. For example, influenza.

**Response***: Thank you for providing us an opportunity to revise our manuscript. In the revised version, we have carefully addressed the questions and comments from the two reviewers. These include performing sensitivity analyses and simulations with irregularly reported data or with time varying reporting rate, clarifying the relationship between our estimation results and the records, and elaborating more details on the application of the proposed model in the complex situations.*

*We are very grateful to you for handling our paper and have followed your suggestion to analyze the transmission heterogeneity of another virus to provide more evidence for the proposed method. Instead of influenza, we chose measles for which the dispersion number was recorded in the classical paper of transmission heterogeneity* [1]*. Our results were consistent with the records in terms of overlapped confidence interval (line 236~240, 347~352).*

**Reviewer #1**:

1) The authors do not formally compare their results to estimates from the same time period and settings, such as Lau 2020. However, the estimated values of k from Lau 2020 are on the order of 10−1 rather than Zhang et al.’s estimates which seem to be somewhere between 10−1 and 10^−2^. Why does this difference occur and does it similarly occur in other settings?

**Response***: Thank you for the insightful comments! The difference between our estimation and that of Lau 2020 was due to the differences both in data sources and in the methods used in the estimation. For example, Lau’s method assumed that the transmission events happened around the residence of the cases and employed their spatial information of to reconstruct the offspring distributions of all cases. As pointed out by the authors, this assumption may “have thus underestimated both the infectiousness of younger adults who are more socially active and the degree of superspreading”. Our method used only the incidence data, and estimated the heterogeneity without assuming where the transmission events happened. So, it is reasonable to get more significant real-time heterogeneity (in terms of smaller dispersion number $k_t$) with our method. Even though, our estimation of overall dispersion number during the whole study period was consistent with with that of Lau’s study. Particularly, our overall estimation of overall heterogeneity was 0.016 (95% HPD: 0.01, 0.023), and Lau’s method generated the overall estimation of 0.009 (95% CI: 0.007, 0.348) [2]. We added these explanations in the manuscript (line 377-383).*

*Our estimation of dispersion number was consistent with those estimation based on contact tracing data. For example, our estimates of the overall dispersion numbers of three epidemics were consistent with the records in literatures. In addition, we estimated the real-time heterogeneity with the incidence data of COVID-19 from 2020-Jan to 2021-Apri in Hongkong China, and compared the estimation with the newly updated research of time-varying heterogeneity on the basis of contact tracing data [3]. Our estimation of real-time dispersion number $k_t$ was fluctuating between 0.1 and 1, which was consistent with those based on the contact tracing data. We added this new result in the subsection of “Time-varying scenario”. This example provided additional evidence for the validity of the proposed method in monitoring transmission heterogeneity (line 397-409).*

2) Incidence data frequently have sources of variation other than due to variation in transmission. How robust is this method to these other sources of variation?

**Response*:*** *Thanks for this important comment! Based on your previous comment, we think that the term of “other sources of variation” in this comment means could for example be irregular reporting. We added an additional simulation study to test the performance of the proposed method in the presence of irregular reporting. As expected, the estimation of dispersion number $k$ became worse with larger estimation bias, lower empirical coverage of 95% HPD and lower probability of correcting identify heterogeneity. These biases came from the fact that irregular reporting would reduce the sample size given the fixed study period and made the rare events being harder to occur in the finite sample.*

*In contrast, the estimation of reproduction number $R$ was less affected, the median of absolute error remained at low level and the empirical coverage 95% HPD remained high.*

*We added these results in the result section (line 207~215, 315~324).*

3) The authors pick very wide “non-informative” priors. Can the sensitivity of the results across a range of prior specifications be checked? Particularly for some the more common types of “weakly informative” priors (see e.g. https://www.mdpi.com/1099-4300/19/10/555/htm)

**Response:** *Thanks for this relevant suggestion! We chose the “non-informative” priors for the parameter of Rt and kt because of the convenience of comparison. Most of the previous studies provided the 95% confidence intervals for the estimation. Our estimation of 95% HPD under non-informative priors could be directly compared with these records in literature.*

*In the future development of our method, one part is clearly to use some informative priors to improve the estimation accuracy. We added this description in the discussion section (line 564~568).*

4) In the simulation study, the results for the dispersion parameter, k, are only reported with respect to its point estimate and if it is above or below 1. Can the authors report the HPD for k and the whether the model appropriately recovers the simulation parameter across the specifications?

**Response:** *Thanks for this helpful suggestion! We computed the empirical coverage of 95% HPD for dispersion number k and reported the simulation study (Figure 1).*

5) Between lines 119 and 120, the equation Λtηt ∼ $\Gamma(k_{t},\frac{k_{t}}{\Lambda_{t}R_{t}})$ seems to imply that Λt is distributed in part according to Λt. Can the authors clarify or modify their notation?

**Response:** *Thanks for this helpful suggestion! Our have now corrected the notation in the manuscript (line 120~121).*

6) The relative RMSE of k is very high when k grows larger. Can the authors explain why this is occurring?

**Response***: Our estimation of dispersion number $k$ was based on its likelihood function. Previous studies showed that the estimation of $k$ on the basis of likelihood function (e.g., the maximum likelihood estimation) was likely to be overestimated from finite sample, especially when the true value of k was larger than 1 (i.e., homogenous condition). This positive bias in k might occur because smaller samples are less likely to include rare extreme values through which the negative binomial distribution manifests its heterogeneity. This also helps to explain why the estimation of $k$ became worse under the condition with irregular reporting where the sample size became even smaller.*

*Fortunately, our estimation performed better when the true value of k was smaller than 1 (i.e., heterogeneous condition). This heterogeneous condition is of more relevant in epidemiological practice compared with the homogeneous condition, so our method is of considerable implication in real applications. We added these comments in the discussion section (line 569~578).*

*Instead of using RMSE, we changed to the criterion of median absolute error (MAD) in the evaluation of the point estimation of $k$ and $R$ since fitting negative binomial distribution is not stable. We revised the criterion in the subsection of simulation and analysis of incidence time series (line 196~200).*

7) The estimated values for k from the Omicron pandemic are astoundingly low – on the order of 10^-4^. Can the authors add additional supplemental detail and discussion on this result, especially in comparison to related values in the literature?

**Response**: *Thanks for the insightful comment! To compare the transmission heterogeneity of the epidemic caused by the SARS-COV-2 variants of Delta and Omicron, we used the proposed method to analyze the incidence curves in South Africa during two periods. We estimated the overall dispersion number of the Delta epidemic was 9.27*10^-4^, and that of the Omicron epidemic was 3.43*10^-4^. The variant of Omicron gained smaller k, suggesting a greater potential for heterogeneous transmission. This finding was consistent with a recent study on the epidemics in South Korea [4].*

*It should be noted that our analysis was based on the incidence data aggregated over the whole nation of South Africa. Our estimation of k represented the comprehensive transmission heterogeneity in this nation and within a large number of infections (about 10^4^ cases per day). Because that the spreading of COVID-19 in such a large population is more complex than those in local areas, our estimates of k were lower than previous studies focusing on some local areas with smaller number of infections [2].*

*The incidence curve may vary with context as various geographic, socioeconomic, and cultural environments may affect the transmission dynamics of infectious disease. Hence our estimation could not be directly used to other epidemics of Omicron. More accurate estimation of transmission heterogeneity of Omicron under various conditions could be obtained with the proposed method if more data are available. To avoid the potential misleading of the estimation result, we added this explanation in the discussion (line 530~533,584~599).*

8) The simulations were only repeated 100 times and the results seem as if they may be noisy (MCSE is high). Can the authors run a much higher number of Monte Carlo simulations?

**Response***: Thanks for this suggestion! We made another simulation study with 500 runs and compared the results with those under the situation of 100 runs (line 327-328 and Figure S3)*

9) While there is code available on Github, it doesn’t seem to have the code to perform the simulation study or produce all of the figures. Only the code for Figures 2, 3, and 4 seems to be available.

**Response***: Thanks for this suggestion! We uploaded all the codes to Github which make our study more transparent to other the researchers.*

10) In general, it’s hard to tell how specifically the simulation study was coded. Was an individual-level branching process generated and then the incidence summed at each time step? Or was incidence generated on the aggregated scale and variance simulated around the mean?

***Response****: Thanks for pointing this out! In our simulation study, we generated the incidence at time t based on the epidemiological parameter (R and k) and the incidence data up to time t-1. In particular, we calculated the total infectiousness at time t as Λt =* $\sum_{s=1}^{t-1} I_{s}w_{t-s}$*, and then generated the incidence I_t_ based on the Negative binomial distribution of I_t_ ~* $NegB(k\Lambda_{t},\frac{k}{k+R})$*. We revised the description in the simulation study and also uploaded the simulation code for your reviewing (line 183~185).*

11) The authors do not report the incidence curves for their simulated results. How many individuals are infected in the simulation and is it comparable to the settings with real data?

***Response****: Thanks! In the simulation study, we started the simulation with 10 infections and continued for 24 days. There were on average about 128, 266, and 600 new infections respectively under the conditions of R = 1.1,1.3, and 1.5. These infection sizes were comparative with those in real datasets. For example, there were totally 135 infections in the COVID-19 outbreak of Tianjing, China in 2020 which was lasting for 25 days (line 292~296). We also added the typical incidence curves under different settings in the supplementary files (Figure S1).*

12) In general, it’s hard to tell from these results when the method is appropriate to apply and when the authors believe it would be inappropriate. Can the authors expand on their limitations to explain when this method should not be applied or when it would fail to perform well?

**Response***: Thanks for this important comment! As demonstrated in the simulation study, our method overestimated the dispersion number especially when its true value is larger than 1. In other words, the method performed worse under the homogenous condition. Also, we found that irregular reporting will affect the estimation. We added these observations in the discussion (line 569~578).*

13) The authors don’t report convergence diagnostics for the Bayesian models.

**Response***: Thanks for this suggestion! We calculated the Effective Sample Size (ESS) for the estimation of dispersion number and reproduction number in the simulation study. In general, the ESS of all the parameters were much high, indicating that the posterior sampling was convergent (line 203~206, 325~326).*

**Minor comments:**

1) In Equation (1), it is unclear in the role of the superscript and the subscript are.

**Response***: The notation of* $\bar{I}_{1}^{t}$ *represented the collection of the incidence data between time 1 and t, which was defined in line 82. So the subscript and the superscript stand for the start and ending time of study. We have revised the description after equation (1) (line 90~91).*

2) I’m not sure what the phrase “evolutionary processes occur on the same timescale as epidemiological processes” (line 26) means.

**Response***:* *Thanks for this question! We wanted to say that the evolution of virus genome at almost the same rate as the transmission of disease, which is the fundamental assumption behind the viral genetic analysis. We revised the description in line 26*

3) Likewise, I’m not sure how to understand “In other words, the standard model is identical to assume $v_{s,t}^{i}=w_{t-s}R_{t}$” (line 115).

**Response***: Thanks for pointing this out! We revised the description in line 116~118.*

4) I also have trouble following lines 465-469.

**Response***: Thanks for pointing this out! In this section, we proposed two possible ways to reduce the delay between the estimation and the true heterogeneity. We have revised the description, now in lines 551-557.*

5) In Figure 3, it’s hard to understand the x axis.

**Response***: Thanks for your careful review! We have revised the notations in the x axis of Figure 3.*

6) In Figure 5, it’s hard to tell what’s happening in the plots of k because the plots are log scaled and the credible intervals are so wide.

**Response***: Thanks for pointing this out! We reset the scope of the y axis in these plots to make the changes more visible. We also added some description in the subsection of sensitivity analysis about how the real-time dispersion number changes with the reporting rate and other biases in the serial interval (line 417~424). We also revised the notations in the x axis as in the Figure 3*

7) On line 379, “The underlying reason for this decrease in Rt was the deploying of control measures by the South Africa government as indicated by the government stringency index.” This statement seems overconfident in ascribing a mechanism behind the decrease.

**Response***: Thanks for pointing this out! We have revised the statement (line 464~466).*

**Reviewer #2**:

1) The authors perform simulation studies to validate their approach. Specifically, they simulated incidence data from their proposed model (a branching process essentially) and showed the key model parameters of interested can be estimated reasonably well. I think this is a good test but sufficient. A much stronger test would be to simulate the epidemic using an individual-level S-I-R type model (a model that is more representative of the real transmission process), and fit their model to the incidence data from the simulation. Of course, parameter like the dispersion parameter like k_t will not longer be explicit in an S-I-R type model, but the authors can compute (and compare) empirical k_t as a summary statistics from the simulated epidemic.

**Response***: Thanks for this valuable suggestion! It would provide stronger evidence of validating the new model with an individual-level S-I-R type model. Similar test was performed on the estimate of real-time reproduction number Rt [5]. The estimate of Rt based on branching process (i.e., Cori’s model) was proven to accurately reveal the true reproduction number. Since our method was also developed on the same framework of branching process model, we speculate that the performance would be similar as under the S-I-R type model.*

*In addition, the extension of simulation study to the SIR type model may lead to some conceptual challenge. For example, there was a lot of discussion on the case reproduction number and instantaneous reproduction number [5]. Similarly, we think there would be corresponding concepts when analyzing transmission heterogeneity. These uncertainties would be beyond the scope of this manuscript but highlight for the next step exploration. We added these comments in the discussion (line 578-583)*

2) I would like to see more in the sensitivity analysis, particularly, on time-varying reporting rate. In its current set of sensitivity analysis, only constant reporting rate is tested. This may be true for pathogens like flu, but I imagine this assumption may break down for novel pathogens like SARS-CoV-2. A time-varying reporting rate will likely to have a large impact on their model estimates, given that their inference largely depends on inputs of incidence data (Equation 4). If this turns out to be case, such sensitivity towards time- varying reporting rate should be reported.

**Response***: Thanks for this valuable suggestion! To consider the effect of time-varying reporting rate, we followed the recent study [6] which showed that the reporting rate increases during the epidemic wave and reaches to a plateau at the end of the epidemic. Therefore, in the Ebola epidemic, we initiated the reporting rate as 0.3, increased to 1.0 at the end of the fourth period (i.e., on 2015-02-09), and remained unchanged till the end (i.e., 2015-03-29). We generated the synthetic incidence time series according to the time-varying reporting rate. By analyzing the synthetic data with the proposed method, we found there is some changes in the estimation of dispersion number. But the temporal trend of dispersion number was not affected too much. We added these results in the sections of method (line 280~285) and result (line 417~424, line 428~431). We also revised the Figure 6.*

3) In contrast to a general spatio-temporal transmission model, their approach considers only the temporal aspect of the transmission. I would like the authors to discuss more on how potential spatial heterogeneity of transmissibility may impact their results, and make related caveats clearer in the discussion.

**Response***: Thanks for this important comment! The spatial heterogeneity was indeed overlooked by our model. We should be cautious about the scope of using our estimation results. In addition, our model provided a new tool of studying how the spatial heterogeneity and the underlying factors affect the transmission dynamics of epidemic at different scales. We added some comment in the discussion section (line 584~599).*

Reference

[1] Lloyd-Smith JO, Schreiber SJ, Kopp PE, Getz WM. Superspreading and the effect of individual variation on disease emergence. Nature. 2005;438(7066):355–359.
[2] Wang J, Chen X, Guo Z, Zhao S, Huang Z, Zhuang Z, et al. Superspreading and heterogeneity in transmission of SARS, MERS, and COVID-19: A systematic review. Computational and Structural Biotechnology Journal. 2021;19:5039–5046.
[3] Adam D, Gostic K, Tsang T, Wu P, Lim WW, Yeung A, et al. Time-varying transmission heterogeneity of SARS and COVID-19 in Hong Kong. 2022.
[4] Guo Z, Zhao S, Ryu S, Mok CKP, Hung CT, Chong KC, et al. Superspreading potential of infection seeded by the SARS-CoV-2 Omicron BA. 1 variant in South Korea. Journal of Infection. 2022;
[5] Gostic KM, McGough L, Baskerville EB, Abbott S, Joshi K, Tedijanto C, et al. Practical considerations for measuring the effective reproductive number, R t. PLoS computational biology. 2020;16(12):e1008409.
[6] Spannaus A, Papamarkou T, Erwin S, Christian JB. Inferring the spread of COVID-19: the role of time-varying reporting rate in epidemiological modelling. Scientific Reports. 2022;12(1):1–12.
